# Introducing trainees to research using an online, asynchronous course

**DOI:** 10.1017/cts.2023.579

**Published:** 2023-06-29

**Authors:** Jason T. Blackard, Jacqueline M. Knapke, Stephanie Schuckman, Jennifer Veevers, William D. Hardie, Patrick H. Ryan

**Affiliations:** 1 Division of Digestive Diseases, Department of Internal Medicine, University of Cincinnati College of Medicine, Cincinnati, OH, USA; 2 Center for Clinical and Translational Science and Training, University of Cincinnati, Cincinnati, OH, USA; 3 Department of Family and Community Medicine, University of Cincinnati College of Medicine, Cincinnati, OH, USA; 4 Department of Pediatrics, University of Cincinnati College of Medicine, Cincinnati, OH, USA; 5 Division of Biostatistics and Epidemiology, Cincinnati Children’s Hospital, Cincinnati, OH, USA

**Keywords:** Research training, medical student, scholarly activity

## Abstract

**Introduction:**

Research is an important aspect of many students’ training. However, formal research training is rarely included in curricula. Thus, we developed an online, asynchronous series of modules to introduce trainees to multiple topics that are relevant to the conduct of research.

**Methods:**

*Research 101* was utilized by first-year medical students and undergraduate students conducting mentored research projects. Students’ knowledge, confidence, and satisfaction were assessed using pre- and post-module surveys with five-point Likert scaled questions, open-ended text responses, and a final quiz.

**Results:**

Pre-module survey results showed that learners felt most confident with the *Conducting a literature search and Race and racism in medicine* modules and least confident with the *Submitting an Institutional Review Board* protocol at UC module. Post-module survey responses were significantly increased compared to pre-module results for all modules and questions *(p* < 0.0001). The response to “The content of this module met my needs” was endorsed across all modules (84.9% “yes” responses). A final quiz of 25 multiple-choice questions was completed by 92 participants who received a median score of 21. Content analysis of open-ended post-module survey responses identified several strengths and opportunities for improvement in course content and instructional methods.

**Conclusions:**

These data demonstrate that significant learning resulted from completion of *Research 101*, as post-module survey scores were significantly higher than pre-module survey scores for all modules and questions. Final quiz scores were positive but also highlighted opportunity for additional trainee learning and will guide evolution of future modules.

## Introduction

Research is an important component of any medical professional’s training and professional development. A 2015 meta-analysis of medical students found that 72% were interested in conducting research and 31% were interested in a career that included research [1]. Students participating in research activities in medical school were 3.55 times more likely to be interested in research as part of their future careers [1]. The 2021 Association of American Medical Colleges (AAMC) Medical School Graduation Questionnaire reported that 83.9% of medical school graduates participated in a research project with a faculty member compared to 69.3% in 2014 and 77.3% in 2017 [2,3]. Overall, 61.2% of 2021 medical school graduates submitted a paper for publication compared to 48.6% of 2017 respondents, and 51.1% planned to participate in research during their careers [3].

Medical trainees may conduct scholarly activities at various times during their training, including summer electives, mandatory curricular activities, extracurricular research activities, and longitudinal research experiences. AAMC core competencies for entering medical students outline competencies such as quantitative reasoning, critical thinking, and written communication [2]. A study by Amgad *et al*. reported that career advancement is a significant motivation for performing research during medical school [1]. While Green *et al.* found that program directors often ranked research experience lower among selection criteria when all specialties were grouped together, research experience was ranked highly in competitive specialties such as plastic surgery, radiation oncology, dermatology, and neurosurgery [4]. DeFranco and Sowa suggest that rigorous hands-on training in the scientific method will aid in the integration of basic science knowledge with clinical decision-making and ultimately enhance patient care [5]. Nonetheless, formal training in the scientific method and the conduct of research is often fragmented across the medical school curriculum. For instance, Stone *et al*. noted that curriculum efforts varied widely across institutions, and research was often buried within the curriculum and not obvious [6].

The Accreditation Council for Graduate Medical Education (ACGME) mandates that residents must participate in scholarly activity prior to the completion of their training [7]. Nearly all residency programs have established guidelines for scholarly activities that align with accreditation requirements; yet, organized, comprehensive research curricula are often lacking [8]. Benefits of resident research exposure have been well described in the literature (reviewed in [9,10]) and include increased lifelong learning, improved patient care, increased satisfaction with training, and higher likelihood of pursuing academic careers. Nonetheless, several potential barriers exist including a lack of mentors, lack of research infrastructure, lack of trainee interest, lack of financial support, the high demand for clinical responsibilities, and the lack of research curricula.

To establish fundamental research skills and fill gaps within training curricula, we previously piloted an online, asynchronous set of modules – called *Research 101* – to introduce medical students to various topics that are germane to the conduct of research [11]. Post-module mean scores were significantly higher than pre-module results for all modules indicating significant learning by completing *Research 101*. Here, we evaluated the use of *Research 101* across a larger, broad spectrum of learners participating in structured research at the University of Cincinnati College of Medicine and compared learning outcomes across participant groups.

## Methods

The creation and pilot study of *Research 101* has been described in detail elsewhere [11]. Briefly, *Research 101* modules were offered asynchronously through the online educational platform Canvas (Salt Lake City, UT). The first module – Getting started with *Research 101* – provided a brief introduction to the course content. Each module consisted of several elements including learning objectives, assignments, a pre-module survey, and a post-module survey. To complete a module, participants completed (1) a pre-module survey before reviewing any of the assignments within a module, (2) all assignments within a module, and (3) a post-module survey at the conclusion of each module. The pre-module survey included questions based on the learning objectives of the particular module with responses provided on a 5-point Likert scale. For instance, “I am confident in my ability to…identify my skills as a mentee/trainee” (learning objective #1 for the *Aligning Expectations* module) or “I am confident in my ability to…describe possible barriers to an effective mentor-mentee relationship” (learning objective #4). The post-module survey included the same questions based on the learning objectives as the pre-module survey and two questions with yes/no/unsure response options: (1) The content of this module met my needs? and (2) Would you recommend this module to a friend if it was not a requirement? Additionally, open-ended text field questions were included: (1) What did you like most about this module?; (2) What did you like least about this module?; and (3) If you could change one thing about this module, what would it be?

All survey data were collected and managed using the Research Electronic Data Capture tool hosted at the University of Cincinnati [12]. Qualitative survey responses were analyzed using an inductive content analysis approach that offers a systematic and objective method for classifying words and phrases into meaningful categories and permits the analyst(s) to discern key ideas from a larger body of text [13]. Changes in module Likert scale scores were assessed by subtracting pre-module Likert scores from post-module scores such that positive differences indicated increased confidence in knowledge regarding the module content. Statistical significance in pre-post scores was tested using a paired *t*-test (SAS Version 9.4). For group comparisons, pooled p-values were reported when the test for equality of variances (Folded F) was > 0.05. When the equality of variances test was < 0.05, the p-value from the Satterthwaite method was reported.

A final quiz consisting of 25 multiple-choice questions with one correct answer per question was required for all participants. Participants had access to all *Research 101* content during the quiz, feedback on incorrect responses was provided, and there was no time limit. The final quiz score was utilized for reporting purposes only and to refine the module content.

The University of Cincinnati Institutional Review Board (IRB) reviewed the study and determined the research qualified as having minimal risk to participants and was exempt from most of the requirements of the Federal Policy for the Protection of Human Subjects.

## Results

During the 2021–22 academic year, 132 individuals were registered for *Research 101*, including 99 first-year medical students, 23 undergraduates or international medical students, and 10 internal medicine residents. Pre-module and post-module survey results are shown in Table 1. Due to program-specific requirements and/or missing data, the number of individuals responding to specific survey questions is not consistent across all modules. Prior to completing the modules, learners were most confident with the *Race and racism in medicine* (4.15 – 4.21) and the *Conducting a literature search* (4.04–4.10) modules and least confident with the *Submitting an IRB protocol at UC* (2.23–2.37) and the *Study design and data analysis basics* (3.13–3.27) modules. Post-module mean scores were significantly increased compared to pre-module scores for all modules and all learning objectives (*p* < 0.0001).

**Table 1.** Pre-module and post-module survey results for the *Research* 101 modules

Module/Question	*N*	Pre-module mean	Post-module mean
**Introduction to research/**I am confident in my ability to… Define research Identify various types of research Recognize the stages of clinical/translational research Identify the steps of the scientific method	107 107 105 105	3.73 3.51 3.14 3.74	4.50* 4.35* 4.26* 4.50*
**Aligning expectations/**I am confident in my ability to… Identify my skills as a mentee/trainee Identify possible expectations of a mentee Identify possible expectations of a mentor Describe possible barriers to an effective mentor-mentee relationship	107 106 106 105	3.53 3.56 3.50 3.55	4.30* 4.31* 4.27* 4.37*
**Identifying a research mentor and a research project/**I am confident in my ability to… Describe the characteristics of your ideal mentor List resources to identify an appropriate mentor and to develop a research project Strategize possible solutions to common barriers to an effective mentor-mentee relationship Understand how to write a clear and concise research project description	105 107 107 105	3.92 3.30 3.55 3.24	4.51* 4.33* 4.35* 4.19*
**Introduction to human subjects research and protections/**I am confident in my ability to… Understand the history of human subjects research Define human subjects research List the requirements for protecting human subjects in research	106 105 104	3.36 3.54 3.29	4.32* 4.37* 4.36*
**Submitting an Institutional Review Board (IRB) protocol at UC/**I am confident in my ability to… List resources for submitting an IRB protocol for review List the components of an IRB submission Understand common issues with IRB submissions and strategize possible solutions List resources for the protection of human subjects, biosafety, and animal care and training on these topics	104 104 103 104	2.23 2.35 2.33 2.37	3.64* 3.70* 3.55* 3.77*
**Conducting a literature search/**I am confident in my ability to… Describe the use of PubMed for literature searches Describe the use of Google Scholar for literature searches List resources for conducting an effective literature search	105 105 105	4.10 4.09 4.04	4.48* 4.47* 4.49*
**Effective writing for publication/**I am confident in my ability to… List the various types of publications Describe the elements of a manuscript Describe an effective abstract List resources for improving ones writing skills	102 104 104 104	3.37 3.34 3.47 3.03	4.27* 4.27* 4.27* 4.22*
**Transparency, rigor, and reproducibility in research/**I am confident in my ability to… Describe the need for transparency and its impact on the research process Describe the need for rigor and reproducibility and their impact on the research process Identify approaches to enhance the transparency, rigor, and reproducibility of your research project	103 103 103	4.03 4.03 3.63	4.51* 4.48* 4.42*
**Study design and data analysis basics/**I am confident in my ability to… List different types of epidemiologic studies Define key characteristics and limitations of cross-sectional studies Define key characteristics and limitations of cohort studies List resources for study design and data analysis	102 101 102 102	3.14 3.27 3.26 3.13	4.14* 4.13* 4.17* 4.18*
**Presenting your summer research/**I am confident in my ability to… Describe elements of an effective poster List venues for presenting research projects Describe elements of an effective lab meeting presentation	103 103 103	3.59 3.14 3.23	4.38* 4.20* 4.17*
**Evaluating the literature and presenting a journal club/**I am confident in my ability to… Describe strategies for reading and critiquing a scientific article Describe elements of an effective journal club	101 101	3.55 3.21	4.25* 4.18*
**Race and racism in medicine**/I am confident in my ability to… Understand race as a social construct Understand historical examples of racism and bias that have impacted research and medicine Appreciate the connection between systemic racism and social determinants of health	102 90 101	4.20 4.21 4.15	4.50* 4.52* 4.49*

*p < 0.0001.

**Figure 1.**
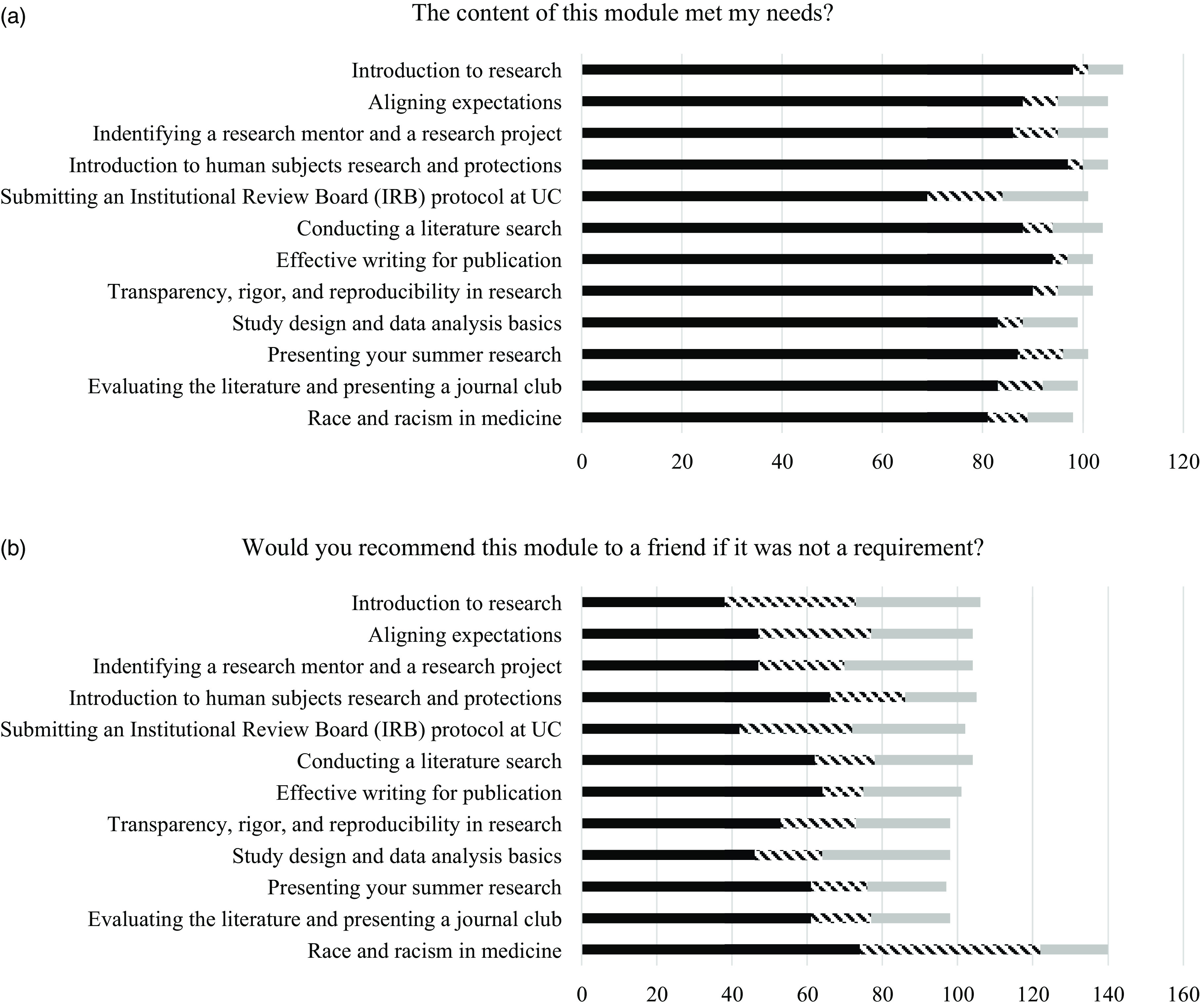
**(** *a* **)** Responses to the question “the content of this module met my needs” and **(** *b* **)** “would recommend this module to a friend if it was not a requirement.” Yes responses are black, no responses are hashed, and unsure responses are gray.

As shown in Fig. 1a, the response to “The content of this module met my needs” was endorsed highly across all modules (84.9% “yes” responses). “No” and “unsure” responses were highest for the *Submitting an IRB protocol at UC* module and lowest for the *Introduction to human subjects research and protection* and *Effective writing for publication* modules. Across all modules, the response to “Would you recommend this module to a friend if it was not a requirement?” received 52.6% “yes” responses, 22.4% “no” responses, and 25.0% “unsure” responses (Fig. 1b). “No” and “unsure” responses were highest for the *Introduction to research* module and lowest for the *Presenting your summer research* module.

Because of potential differences across learner types, we compared findings from the two largest groups of participants, namely medical students and undergraduate students. Several differences in the magnitude of learning – as measured by the average change in survey scores (post-module – pre-module) by group – were observed (Table 2). Each showed a higher magnitude of change among undergraduate students compared to medical students. For instance, the change in average score was higher for undergraduate students compared to medical students for the “identify the steps of the scientific method” (1.32 versus 0.60; *p* < 0.001). Similarly, the change in average score was higher for undergraduate students compared to medical students for the “understand how to write a clear and concise research project description” (1.65 versus 0.79; *p* = 0.001). Overall, the average change in score was higher for undergraduates than for medical students for 17 of 41 (41.5%) learning objectives.

**Table 2.** Average change in Likert scores (post-pre) by undergraduate versus medical student status

Module/learning objective	Undergraduate (*n* = 19)	Medical student (*n* = 97)	P value*
**Introduction to research**			
Define research	1.05	0.69	0.052
Identify various types of research	0.95	0.73	0.226
Recognize the stages of clinical/translational research	1.68	0.87	0.000
Identify the steps of the scientific method	1.32	0.60	0.000
**Aligning expectations**			
Identify my skills as a mentee/trainee	0.89	0.72	0.413
Identify possible expectations of a mentee	0.83	0.69	0.618
Identify possible expectations of a mentor	0.94	0.74	0.477
Describe possible barriers to an effective mentor-mentee relationship	0.82	0.77	0.799
**Identifying a research mentor and a research project**			
Describe the characteristics of your ideal mentor	0.61	0.58	0.870
List resources to identify an appropriate mentor and to develop a research project	1.33	0.95	0.167
Strategize possible solutions to common barriers to an effective mentor-mentee relationship	1.06	0.72	0.100
Understand how to write a clear and concise research project description	1.65	0.79	0.001
**Introduction to human subjects research and protections**			
Understand the history of human subjects research	1.35	0.86	0.036
Define human subjects research	1.24	0.74	0.018
List the requirements for protecting human subjects in research	1.44	1.01	0.085
**Submitting an Institutional Review Board (IRB) protocol at UC**			
List resources for submitting an IRB protocol for review	1.75	1.33	0.141
List the components of an IRB submission	1.63	1.26	0.186
Understand common issues with IRB submissions and strategize possible solutions	1.56	1.17	0.154
List resources for the protection of human subjects, biosafety, and animal care and training on these topics	1.69	1.35	0.204
**Conducting a literature search**			
Describe the use of PubMed for literature searches	0.63	0.32	0.000
Describe the use of Google Scholar for literature searches	0.44	0.34	0.529
List resources for conducting an effective literature search	0.69	0.39	0.205
**Effective writing for publication**			
List the various types of publications	1.40	0.77	0.016
Describe the elements of a manuscript	1.56	0.82	0.008
Describe an effective abstract	1.00	0.75	0.266
List resources for improving ones writing skills	1.75	1.07	0.006
**Transparency, rigor, and reproducibility in research**			
Describe the need for transparency and its impact on the research process	0.94	0.39	0.035
Describe the need for rigor and reproducibility and their impact on the research process	0.75	0.37	0.035
Identify approaches to enhance the transparency, rigor, and reproducibility of your research project	1.13	0.72	0.109
**Study design and data analysis basics**			
List different types of epidemiologic studies	1.63	0.87	0.002
Define key characteristics and limitations of cross-sectional studies	1.75	0.67	0.002
Define key characteristics and limitations of cohort studies	1.63	0.71	0.000
List resources for study design and data analysis	1.56	0.95	0.012
**Presenting your summer research**			
Describe elements of an effective poster	0.71	0.78	0.726
List venues for presenting research projects	1.06	1.08	0.926
Describe elements of an effective lab meeting presentation	0.88	0.93	0.864
**Evaluating the literature and presenting a journal club**			
Describe strategies for reading and critiquing a scientific article	0.88	0.63	0.287
Describe elements of an effective journal club	1.31	0.85	0.086
**Race and racism in medicine**			
Understand race as a social construct	0.56	0.26	0.165
Understand historical examples of racism and bias that have impacted research and medicine	0.85	0.29	0.006
Appreciate the connection between systemic racism and social determinants of health	0.63	0.28	0.046

*Pooled *P* values were used when the test for equality of variances (Folded *F*) was > 0.05. When the equality of variances test was < 0.05, the p value from the Satterthwaite method was reported. *P* values < 0.05 are highlighted in blue.

**Figure 2.**
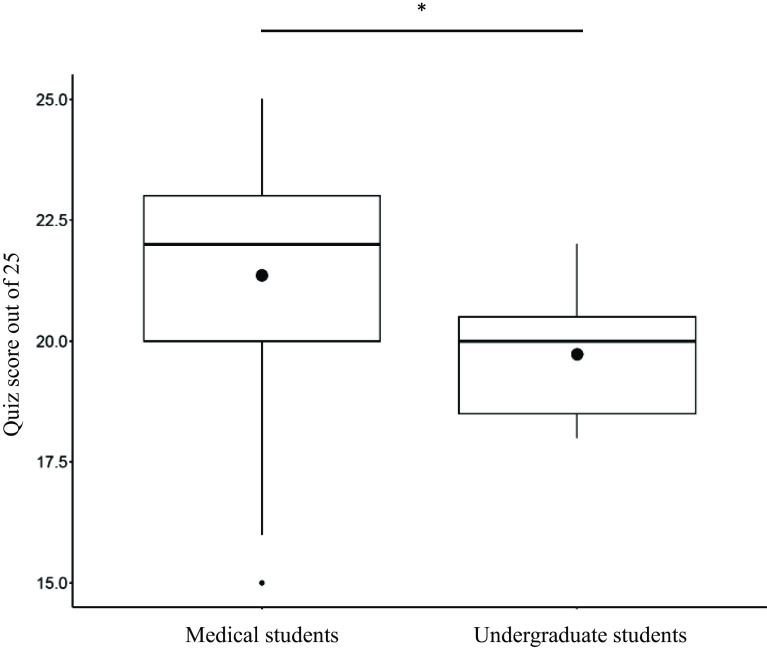
Final quiz scores (of a possible 25) for 81 medical students and 11 undergraduate students. (21.4 versus 19.7; *p* = 0.0072).

The final quiz was completed by 92 participants, including 81 medical students and 11 undergraduate students. As shown in Fig. 2, the average score out of a possible score of 25 for medical students was higher than for undergraduate students (21.4 versus 19.7; *p* = 0.0072).

Content analysis of the open-ended text responses gathered from the post-module surveys revealed several strengths of the *Research 101* modules. First, students appreciated videos that (1) were succinct, easily understood, and visual, (2) presented diverse opinions by dynamic speakers, and (3) struck an appropriate balance between introductory level for basic topics and more comprehensive level for topics that required detailed information. For instance, a participant stated “[This module] covered important topics in engaging way with short videos.” A second strength that participants identified was case studies that demonstrated real-world scenarios. “Case studies highlighted real potential problems one might encounter entering a research setting.” A third strength was the practical and relevant information presented in the form of examples (e.g., IRB, past projects, stages of CTR research, peer review process, mentoring relationships, misconduct, journal clubs), activities that allowed participants to apply their learning to their interests and work (e.g., finding an article, writing clearly and concisely, reading scientific articles, reflecting on their research question, designing a poster), and resources (e.g., Google Scholar, PubMed, NIH RePORTER) that could be referenced later. Participants stated “[This module] discussed a very important topic and gave good examples” and “I like having these resources available for if I ever need them in the future.” Many students appreciated examples that were specific to our institution (e.g., UC Radiation Study, UC Libraries, UC IRB), which discussed difficult topics, such as racism and human subjects’ violations, and historical examples that were particularly impactful, primarily with regard to research ethics. “I appreciated the attention giv[en] to Cincinnati’s own involvement in unethical research practices.” Finally, participants noted a strength in the diversity of speakers, viewpoints, and topics.

Qualitative data also identified several suggestions for improvement with respect to *Research 101* content, including videos that were too long (“Could be more concise”), modules with too many links, resources, or discussion board questions (“[I did not like] all of the separate links, just lots of clicking and navigating”), repetition within *Research 101* and/or redundancy with other medical school curricular components (“It is information I was mostly familiar with before”), content that was too introductory or broad to be useful (“It was too broad to develop a good understanding of the material”), need for better examples and more resources that are more diverse in discipline and setting, more concrete and real-world, more current, and/or more practical (“It talked about posters and lab presentations--I wish it also discussed other ways of communicating research”). Some students felt that certain modules were missing information, including how to get funding, opportunities to present research, poster formatting, clinical trial phases, writing a research project description, and overview of the IRB process (“I wish the module addressed ways to bring forward complaints and frustrations in a productive way,” “I wish there was a video on how to find opportunities to present research,” “I wish there was more conversations on how to get funding,” “I wish they went over the different labels of clinical trials [phases]”). Students were mixed on the use of discussion boards; however, the majority would like fewer assigned and preferred to respond to each other rather than the main thread (“[I did not like having] 4 discussion boards in 1 module”). Technical issues (e.g., nonfunctioning links, audio problems, or the need to add closed captioning to videos) were a minor theme.

## Discussion

Research training is a cornerstone of education and professional development, and resources to provide this training in a comprehensive, student-centered manner are highly sought after. Unfortunately, significant barriers exist to pursuing research training during medical school, such as lack of infrastructure, a paucity of high-quality faculty mentors, insufficient institutional incentives for those conducting research, limited awareness of local research opportunities, and the absence of a research office or coordinator for training [14,15]. In a survey of US medical students, 19.4% reported taking a required course on research methods, and 28.7% reported that a research elective was available at their institution [15]. Structured training in research is important for residency programs as well and is included in current ACGME requirements [7]. It has been suggested that resident research may improve clinical care by fostering clinical evaluation skills, clinical reasoning, and lifelong learning [16,17]. Early exposure to research [18] may also increase the number of physician scientists [17,19]. Furthermore, residency training programs with organized programs/curricula, including protected time for research, were associated with increased productivity [20]. Yet, there is limited information on what specific topics should be taught and existing curricula may not be readily available to those interested in modifying them for their own programmatic needs [8,17]. A systematic review of research curricula for residents found that the most common objectives were to increase research productivity and to enhance critical evaluation skills [17].

*Research 101* was developed to provide a structured introduction to important topics in research in a highly accessible format. Its online, asynchronous format offers a basic training infrastructure that is sufficiently flexible to enable individualized learning and/or program-specific adaptations. Evaluating the expansion of *Research 101* to include other learner types demonstrated significant learning by completing *Research 101* (i.e., post-module survey scores were significantly higher than pre-module scores for all modules and all learning objectives). In general, final quiz scores were high; however, mean scores were different for medical students compared to undergraduate students highlighting the opportunity for additional learning by all future participants enrolled in *Research 101*.

This study has several limitations to consider. First, *Research 101* was piloted initially with a small number of medical students and subsequently expanded to include additional training programs and learner types. Different educational needs based on learner type are likely, and modules may need to be tailored to these specific needs in the future when more participant data have been gathered. Second, individual learners may complete a different set of modules based on their programmatic requirements (i.e., not all participants complete all modules). Third, selection bias may exist among the students who completed *Research 101*. Those that participated may be inclined to have research interests or had previous research experience prior to starting *Research 101* when compared to their nonparticipant counterparts. Nonetheless, the qualitative data that are collected for each module are helpful for regular enhancement of *Research 101* for future audiences that may have limited exposure to scholarly activities. Fourth, while Research 101 is offered asynchronously, some topics may require more direct interaction with learners. Thus, it is important to highlight that *Research 101* should not replace in-person interactions; rather, it provides an additional option for learners given their distinct learning styles, limited space for new content within existing medical school curricula, and the varying interests and backgrounds of learners.

In conclusion, *Research 101* is a valuable addition to the research training toolkit that can be utilized by distinct learner types and result in clear learning. *Research 101* can bolster current training and/or fill existing gaps within undergraduate and medical school degree programs.
